# Investigation of the potential role of fusicoccin, a fungal phytotoxin, in mitigating salt stress in onion roots

**DOI:** 10.1038/s41598-023-36917-4

**Published:** 2023-06-16

**Authors:** Kürşat Çavuşoğlu, Dilek Çavuşoğlu

**Affiliations:** 1 Faculty of Arts and Science, Department of Biology, Süleyman Demirel University, Isparta, Turkey; 2 Atabey Vocational High School, Department of Plant and Animal Production, Isparta University of Applied Sciences, Isparta, Turkey

**Keywords:** Plant cell biology, Plant physiology

## Abstract

Fusicoccin is a diterpene glycoside that plays an important role in the regulation of plant growth and development. Fusicoccin produced by *Fusicoccum amydali* fungus is known to affect plant growth positively with external applications due to its potential to stimulate the tolerance system of plants under stress conditions. In this study, it was aimed to reduce the negative effects of salt (0.15 M NaCl) stress on the germination and growth of onion (*Allium cepa* L.) bulbs by external fusicoccin (3 µM) application. For this purpose, the germination percentage, root length, root number, fresh weight, mitotic activity, micronucleus frequency, chromosomal abnormality, antioxidant enzyme activity, osmolyte accumulation, cell membrane damage and root anatomical structure were investigated in the current study. Salt stress caused a statistically significant difference (p < 0.05) in all examined parameters. External application of fusicoccin to onion bulbs germinated under salt stress conditions was found to be promising as a plant growth promoter and mitosis stimulator. In addition, fusicoccin application alleviated the harmful effects of salt stress on the chromosome structure and root anatomical structure and protected the cells from the cytotoxic and genotoxic effects of salt. Moreover, this application contributed to the fight against reactive oxygen species of onion plant and increased salt tolerance by regulating the accumulation of osmolyte substances such as proline and antioxidant enzymes such as superoxide dismutase and catalase, and by minimizing cell membrane damage in root cells. In conclusion, this study showed that exogenous application of 3 µM fusicoccin reduced the damage caused by oxidative stress in onion bulbs and served for healthy germination and growth.

## Introduction

Salinity is one of the most important environmental stress factors affecting crop production and productivity^1,2^. In arid and semi-arid climatic regions, approximately 23% of agricultural lands are affected by high salinity due to insufficient precipitation and high evaporation, insufficient drainage, improper agricultural practices and artificial irrigation methods^3^. Plants are extremely sensitive to salt stress and can exhibit many differences in salinity at the morphological, anatomical, physiological, cytogenetic and biochemical levels^4–6^. In addition, salinity increases the formation of reactive oxygen species (ROS) in plants and causes both osmotic and oxidative stresses. The resulting ROS cause lipid peroxidation, antioxidant enzyme inactivation, DNA damage and protein denaturation in the membranes of plant cells^7–9^.

Plants can reduce osmotic and oxidative stress damage caused by salinity and provide salinity tolerance by activating some basic mechanisms such as osmoregulation, ion separation and up-regulation of antioxidant activities^10,11^. In addition, they can reduce and repair the damages of salinity stress by increasing the synthesis of antioxidant enzymes such as catalase (CAT) and superoxide dismutase (SOD) and non-enzymatic antioxidants such as ascorbate, salicylate, glutathione and carotenoids^12–14^.

The term phytotoxin is used by plant pathologists to refer to highly toxic substances produced by plants and plant pathogens. The terms plant poisons, plant allelochemicals and phytochemicals are also frequently used to express these substances that belong to the group of plant secondary metabolites^15,16^. These toxins occur naturally in both vegetative (leaf, bark and root) and generative (flower and fruit) organs of plants^17,18^. Phytotoxins are considered as plant growth regulators because they exert their stimulating effects on plants at very low concentrations^19,20^. They also allow to grow and survive of plants under various biotic and abiotic stress conditions^21,22^.

Phytotoxin fusicoccin (FC) is a diterpene glycoside produced by the fungus *Fusicoccum amydali*, a parasite of almond and peach trees^23^. FC has an original carbon skeleton characterized by a 5–8–5 ring system shared with other bioactive terpene families such as ophiobolins and cotylenes^24^. FC has a detrimental effect on plants and causes their death. Both the remarkable phytotoxic activity of FC and the fact that it is even more effective than the hormone auxin in stimulating cell and tissue growth has led to further research on its effects in plants^25^. Although the mode of action of FC at the molecular level is different, in many respects it mimics the effects of the hormone auxin^23^. It is well known that FC binds to the H^+^-ATPase/14–3–3 complex, stabilizing it, thereby causing an increase in H^+^ pump efficiency^26^. In addition, this toxin stimulates various physiological and biochemical processes such as cell wall acidification, cell elongation, breaking seed dormancy, ethylene production, stomatal opening, nutrient uptake, cytochrome c release from mitochondria, solute transport, seed germination, seedling growth, chlorosis, necrosis and transpiration in plants^25,27–29^.

*Allium cepa* L. (onion), belonging to the *Amaryllidaceae* family and the *Allioideae* subfamily, is considered one of the most consumed and grown vegetables in the world^30^. In addition to its nutritional value, it has been reported to be used as a medicinal plant since ancient times due to its antioxidant, antidiabetic and antimicrobial effects^31^. Mostly a biennial or perennial herb, *Allium cepa* L. can grow to about half a meter. Its bulb can be spherical, oval or elongated. Its body is cylindrical and hollow. The leaves can be yellowish to bluish green. They are fleshy, hollow, cylindrical and flat on one side. Seeds are shiny black. Its flowers are hermaphrodite and greenish-white^32,33^. On the other hand, it is preferred as a bioindicator in experimental researches for different reasons such as its easy supply and cultivation, low number of chromosomes and large chromosomes, and easy measurement of biochemical products such as enzymes and proteins^34^.

Studies on exogenous FC application during the germination stage of *Allium cepa* L. bulbs under saline conditions are not available in the literature. Therefore, in this study, it was aimed to alleviate or eliminate the inhibition of salt stress on various parameters (physiological, cytogenetic, biochemical and anatomical) in onion plants germinated in saline conditions with FC.

## Materials and methods

### Determination of experimental material and effective concentrations

This study was carried out with bulbs of onion (*Allium cepa* L.) grown as an important agricultural plant. Bulbs were purchased from a commercial store in Isparta/Turkiye. Fusicoccin/FC (CAS number: 20108-30-9) was supplied from Sigma-Aldrich Company, and sodium chloride/NaCl (CAS number: 7647-14-5) was supplied from Merck Company. As a result of a preliminary experiment conducted by us, the NaCl stress level to which the bulbs are exposed was determined as 0.15 M, and the externally applied FC dose was determined as 3 µM. Experimental research on plant samples, including the supply of plant material, complies with institutional, national and international guidelines and legislation.

### Growing conditions and experimental plan

**Figure 1:**
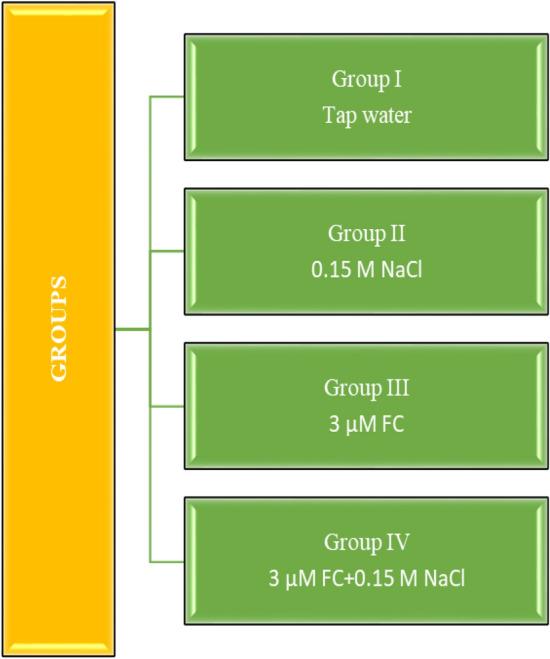
Experimental groups. *FC* fusicoccin, *NaCl* sodium chloride.

**Figure 2:**
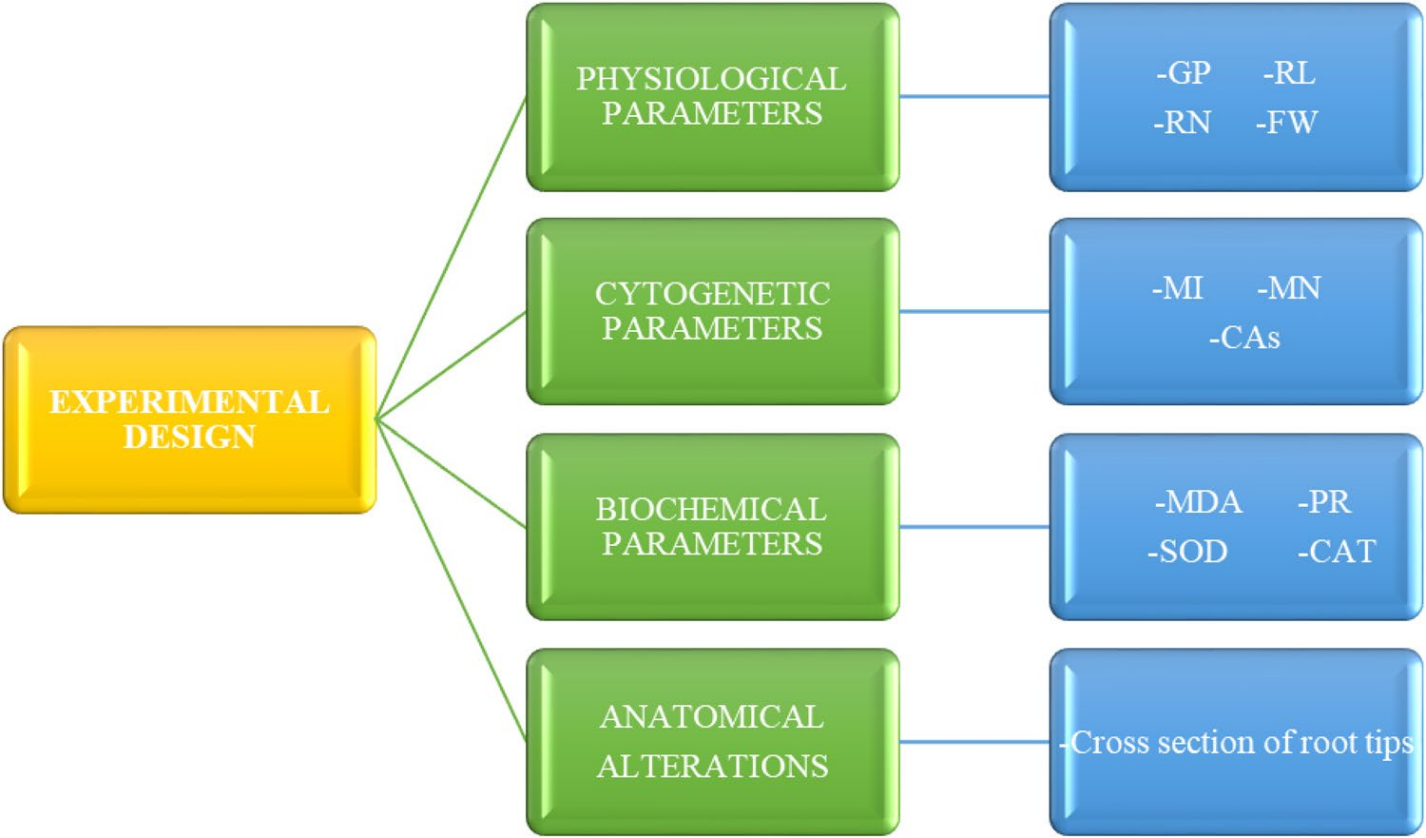
Experimental stages of the study. *GP* germination percentage, *RL* root length, *RN* root number, *FW* fresh weight, *MI* mitotic index, *MN* micronucleus, *CAs* chromosomal abnormalities, *MDA* malondialdehyde, *PR* proline, *SOD* superoxide dismutase, *CAT* catalase.

**Figure 3:**
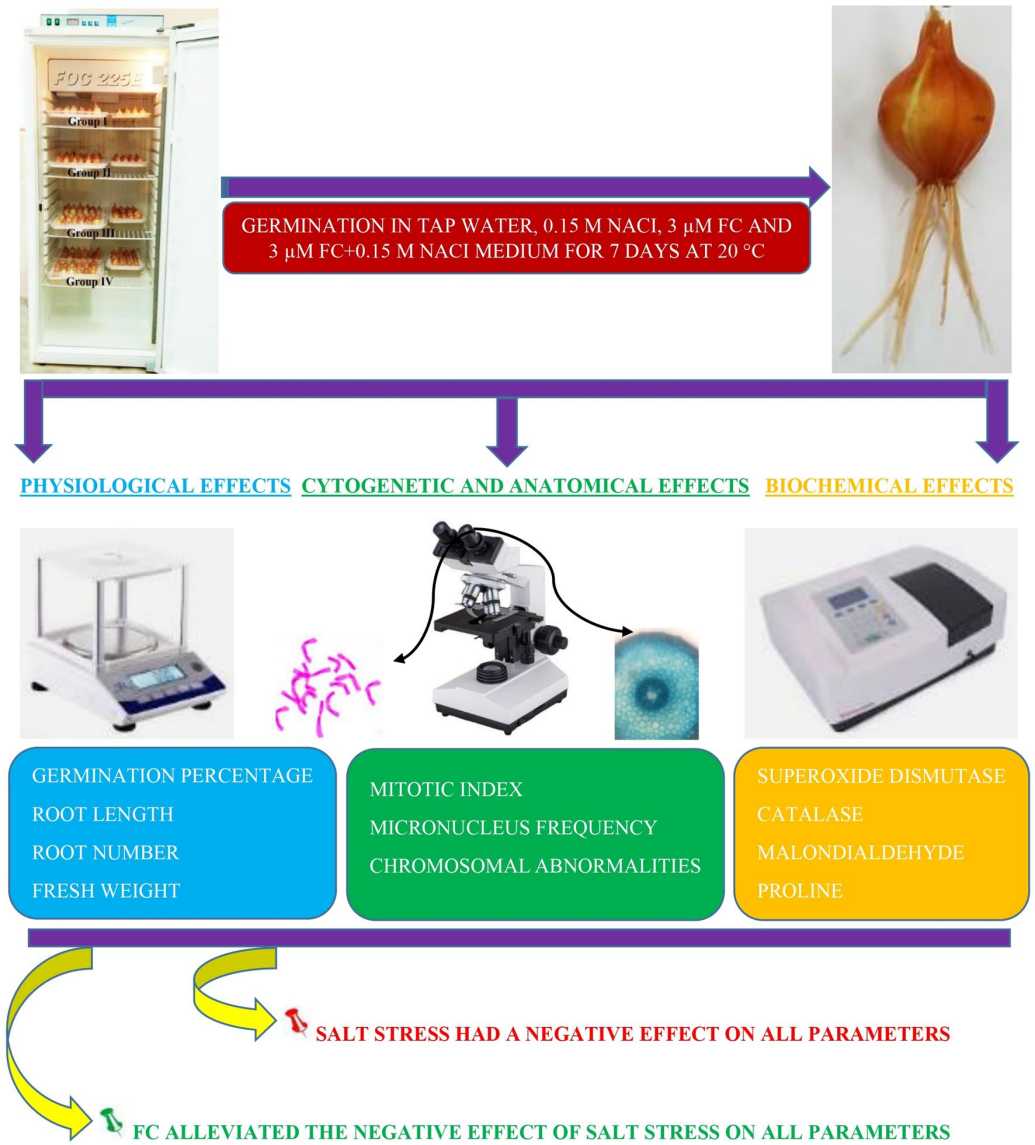
Schematic summary of the study. *FC* fusicoccin, *NaCl* sodium chloride.

Approximately the same size, plump and healthy bulbs were selected. Bulbs separated into four groups. The groups, experimental stages and schematic summary of the study are shown in Figs. 1, 2 and 3. 20 bulbs of each determined group were placed in sterile plastic containers with a volume of 1.7 L and with a perforated lid on which the bulbs will enter, with the root parts inside and the other parts outside, and germinated in the dark for 168 h (7 days) in an incubator set at 20 °C. Control bulbs in the first group were germinated in tap water medium until the end of the study; the bulbs in the second group were germinated in salt stress (0.15 M NaCl) medium; bulbs in the third group were germinated in FC (3 µM) medium; the bulbs in the fourth group were germinated in FC treated medium together with salt stress (0.15 M NaCl + 3 µM FC).

At the end of the 168 h application period, the hairy roots of the germinated bulbs belonging to the control and treatment groups were counted; root lengths were measured in mm by using a ruler with millimetric scales; fresh weights were determined in g by using precision balance, and germination percentages were expressed in % with the help of Eq. (1). The protrusion of the radicle from the testa by 10 mm was taken as the germination criterion. All trials in this study were arranged in triplicate in order to statistically interpret the data obtained^35^.

1 $$ {\text{Germination }}\left( \% \right) \, = \, \left[ {{\text{number}}\;{\text{ of }}\;{\text{germinated }}\;{\text{bulbs}}/{\text{total }}\;{\text{number}}\;{\text{ of}}\,{\text{ bulbs}}} \right] \, \times { 1}00 $$

### Determination of mitotic index and chromosomal abnormalities

In order to detect chromosomal damage, materials cut about 1–2 cm long from the onion root tip were taken into saturated paradichlorobenzene for 4 h, fixed in 3 parts of ethyl alcohol: 1 part of acetic acid solution and stored in 70% ethyl alcohol. For permanent preparation processes, root tips were hydrolyzed in 1 N HCl at 60 °C for 17 min, stained with Feulgen for 1–1.5 h, crushed on a slide in 45% acetic acid, covered with a coverslip, balm applied around the coverslip, and photographed under a microscope at 100 × magnification^36^. In order to calculate the mitotic index (MI), 30.000 cells were counted for each root tip from the prepared preparations and the percentage of cells entering mitosis was calculated using Eq. (2). Chromosomal abnormalities (CAs) were calculated as % of 2.000 dividing cells counted.

2 $$ {\text{MI}}(\% ) \, = \, [{\text{number }}\;{\text{of }}\;{\text{cells }}\;{\text{undergoing }}\;{\text{mitosis}}/{\text{total}}\;{\text{ number}}\;{\text{ of }}\;{\text{cells}}] \, \times {1}00 $$

### Spectrophotometric determination of antioxidant enzyme activity

An amount (0.2 g) of root sample was homogenized with 50 mM chilled sodium phosphate buffer (pH 7.8). The homogenate was filtered through coarse filter paper and centrifuged at 10,000 rpm for 20 min. The supernatant was used for the spectrophotometric analysis of superoxide dismutase (SOD) and catalase (CAT) enzyme activities.

SOD activity; it was determined by examining the photochemical reduction of NBT (Nitroblue tetrazolium chloride) at 560 nm. The reaction was performed in a total of 1.5 mL of 0.05 M sodium phosphate buffer (pH 7.8) containing 750 μM NBT, 130 mM l-methionine, 0.1 mM EDTA-Na_2_, 20 μM riboflavin, 4% polyvinylpyrrolidone, supernatant and deionized water. Riboflavin was added last in the dark and the reaction mixture was incubated for 10 min under 15-W fluorescent light^37^. SOD activity was expressed as “U/mg FW”^38^.

CAT activity; it was measured by tracking the decrease in absorbance at 240 nm. The reaction was carried out by incubating 0.1 M H_2_O_2_, supernatant and deionized water at 37 °C for 2 min in 200 mM pH 7.8 sodium phosphate buffer, and the reaction was terminated with 1 M HCl. One unit of enzyme activity was defined as the amount of enzyme required for the degradation of 1 μmol H_2_O_2_. CAT activity was expressed as “OD240 nm min/g FW”^39^.

### Spectrophotometric determination of lipid peroxidation

Lipid peroxidation is expressed as malondialdehyde (MDA) content. 0.5 g root sample taken from onion roots was homogenized with 10 mL of 5% trichloroacetic acid (TCA), and then the homogenate was centrifuged at 12,000 rpm at 24 °C for 15 min. 1 mL of the clear part of the centrifuged sample was taken and 0.5% thiobarbituric acid (TBA) dissolved in 4 mL of 20% TCA was added to it. After the mixture was kept at 96 °C for 25 min, it was rapidly cooled in an ice bath and centrifuged at 10,000 rpm for 5 min. Then, absorbance was determined at 532 nm wavelength from the clear part and MDA content was calculated by using the extinction coefficient of 155 M^−1^ cm^−1^ and expressed as “µmol/g FW”^40^.

### Spectrophotometric determination of free proline amount

Fresh root sample (0.5 g) was homogenized with sulfosalicylic acid (10 mL of 3%). Root samples were then passed through Whatman filter paper. 2 mL of extract was taken, 2 mL of acid anhydrin and 2 mL of glacial acetic acid were added. The mixture was first kept in a 100 °C water bath for 1 h and then in an ice bath for 5 min. Toluene (5 mL) was added to the reaction mixture, mixed with vortex for 15–20 s, and left for a while to form two phases. The upper phase was taken with the help of a micropipette and the absorbance values were read in the spectrophotometer at 520 nm against the pure toluene control. The results from the examples were compared with the results from the L-proline standard. The amount of free proline (PR) was calculated with the help of Eq. (3) and expressed as “µg/g”^41^.

3 $$ \left[ {\left( {\upmu {\text{g}} \,  {\text{proline }}/{\text{ mL }} \times {\text{ mL}}\;{\text{ toluene}}} \right) \, /{ 115}.{5 }\upmu {\text{g }}/ \, \upmu {\text{mole}}} \right] \, / \, \left[ {\left( {{\text{g }}\;{\text{sample}}} \right) \, /{5}} \right] \, = \, \upmu {\text{moles}}\;{\text{ proline}}/{\text{g }}\;{\text{of }}\;{\text{fresh }}\;{\text{weight }}\;{\text{material}} $$

### Microscopic analyzes

For the detection of anatomical damage, cross-sections were taken from the root tips of the bulbs treated with FC and NaCl for 168 h, stained with 2% methylene blue, closed with the help of entellan, and photographed at 500 × magnification.

### Data analysis

Statistical analyzes of the obtained data were made by using the SPSS 23 analytical software program for Windows and the differences between the results were presented as mean ± standard deviation. It was analyzed at p < 0.05 significance level by using one-way ANOVA followed by Duncan test.

### Ethics approval and consent to participate

The authors confirm that the manuscript has been read and approved by all authors. The authors declare that this manuscript has not been published and not under consideration for publication elsewhere.

## Results and discussion

### Effect of FC on the physiological parameters

**Figure 4:**
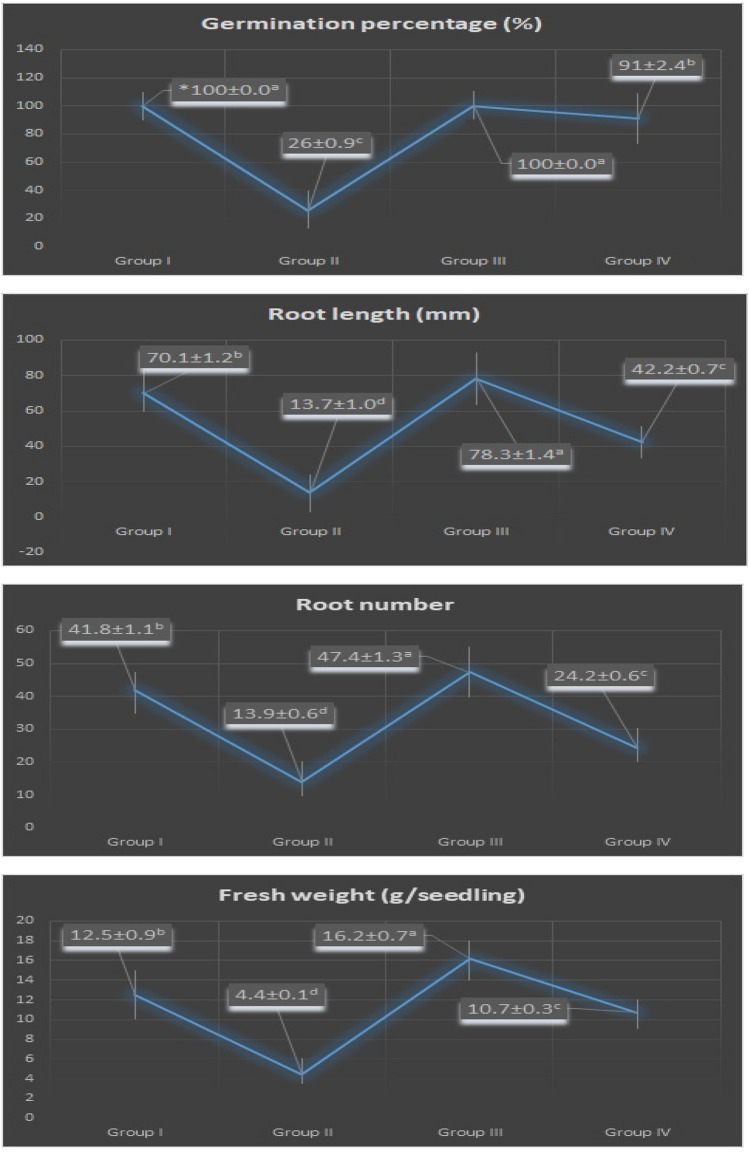
Effect of FC on some physiological parameters of *Allium cepa* L. Group I (control) was treated with tap water; Group II was treated with 0.15 M NaCl; Group III was treated with 3 µM FC; Group IV was treated with 3 µM FC + 0.15 M NaCl. The error bars indicate the standard deviation (± SD).

Figure 4 shows the effect of exogenous FC on the physiological parameters of *Allium cepa* L. bulbs. While external FC application showed the same effect (100 ± 0.0%) as the control group on the germination of onion bulbs under normal conditions, it showed an encouraging effect on the growth parameters of the bulbs. Namely, the root length (RL), root number (RN) and fresh weight (FW) of Group I (control) bulbs grown in tap water medium were 70.1 ± 1.2 mm, 41.8 ± 1.1 and 12.5 ± 0.9 g, respectively, while these parameters were determined as 78.3 ± 1.4 mm, 47.4 ± 1.3 and 16.2 ± 0.7 g in Group III bulbs grown in medium with FC alone. Some researchers reported that exogenous FC application severely caused inhibition of the seed germination and early seedling growth in *Cercis siliquastrum*^42^ and *Pisum sativum*^43^ under normal conditions. On the contrary, some researchers also determinated that FC treatment significantly stimulated the seed germination and seedling growth in *Raphanus sativus*^28^, *Allenrolfea occidentalis*^44^, *Ceratoides lanata*^45^ and *Sorghum vulgare*^46^ under non-stress conditions. All these results revealed that exogenous FC showed different effects on seed and bulb germination under stress-free conditions, depending on plant species, pretreatment form and application dose.

**Figure 5:**
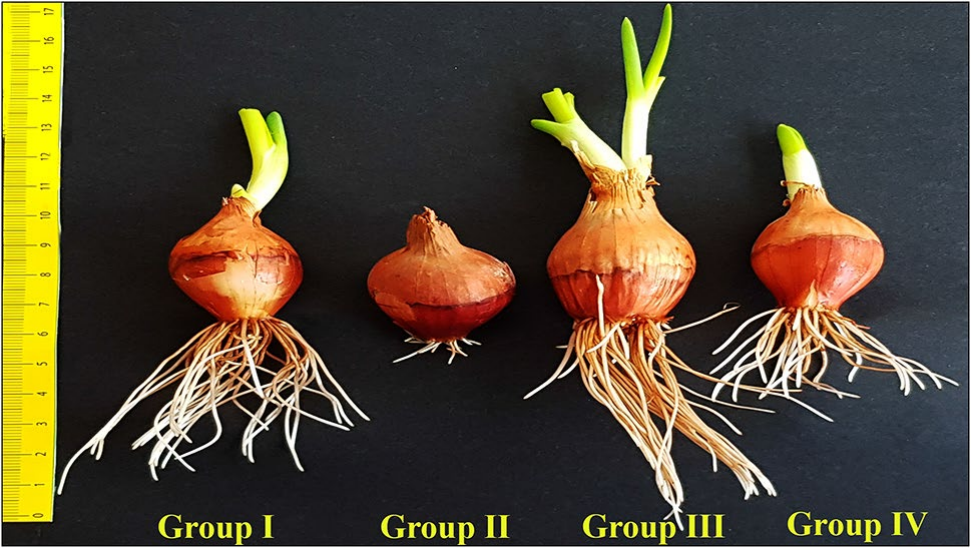
The germination situations at the end of seventh day of *Allium cepa* L. bulbs. Group I was treated with tap water, Group II was treated with 0.15 M NaCl, Group III was treated with 3 µM FC, Group IV was treated with 3 µM FC + 0.15 M NaCl.

**Figure 6:**
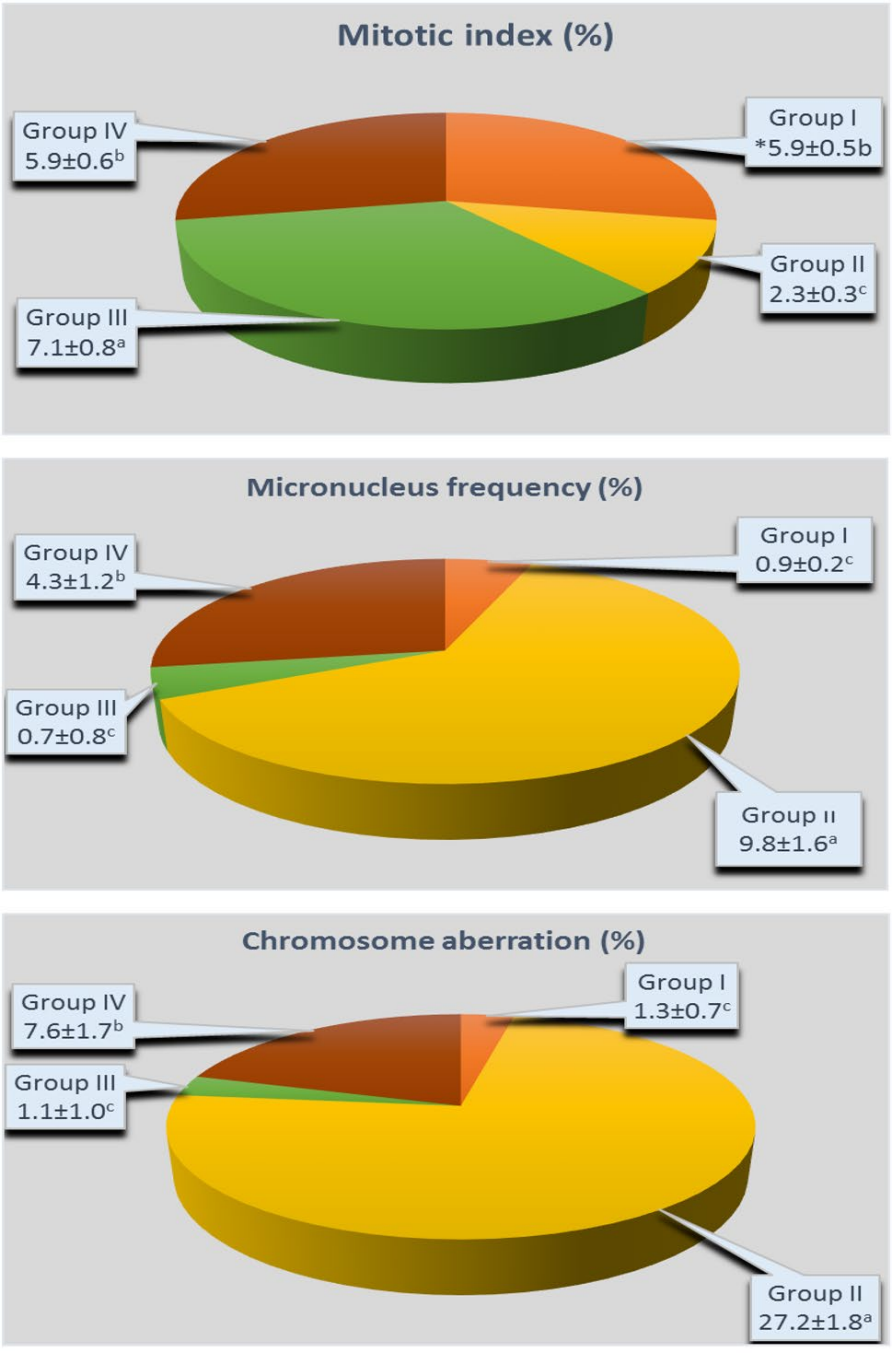
Effect of FC on some cytogenetic parameters of *Allium cepa* L. Group I (control) was treated with tap water; Group II was treated with 0.15 M NaCl; Group III was treated with 3 µM FC; Group IV was treated with 3 µM FC + 0.15 M NaCl. The ± symbol indicate the standard deviation (SD).

Salinity is an important abiotic stress factor that can cause serious damage even to halophytes^47^. In this study, salt stress once again showed its inhibitory effect on the germination and growth of the onion plant (Fig. 4). All of Group I (control) bulbs germinated (100 ± 0.0%) at 168 h (7 days) in tap water medium. However, only 26 ± 0.9% of Group II bulbs germinated in 0.15 M NaCl medium. Therefore, the germination percentage (GP) decreased by 74% in saline conditions. Similarly, RL, RN and FW of Group I (control) bulbs grown in tap water medium were measured as 70.1 ± 1.2 mm, 41.8 ± 1.1 and 12.5 ± 0.9 g, respectively, while Group II bulbs grown in 0.15 M NaCl medium showed a statistically significant decrease (p < 0.05) and was determined as 13.7 ± 1.0 mm, 13.9 ± 0.6 and 4.4 ± 0.1 g (Figs. 4 and 5). The inhibitory effect of salt stress on bulb germination^48,49^ and growth^50,51^ has also been reported in previous studies. Salinity stress may have exerted its negative effects on the growth parameters by affecting various metabolic processes. First, because of the high osmotic pressure, the roots cannot get enough water, and the fresh weight and water content of the bulbs decrease (Fig. 4). Second, salinity inhibits mitotic activity in root tip meristem cells (Fig. 6) and the root number and length of bulbs reduce.

3 µM exogenous FC application to Group IV bulbs germinated in 0.15 M NaCl medium significantly increased GP, RL, RN and FW (p < 0.05). The GP, RL, RN and FW of Group II bulbs in 0.15 M NaCl medium alone were 26 ± 0.9%, 13.7 ± 1.0 mm, 13.9 ± 0.6 and 4.4 ± 0.1 g, respectively, while these parameters were determined as 91 ± 2.4%, 44.2 ± 0.7 mm, 24.2 ± 0.6 and 10.7 ± 0.3 g in Group IV bulbs in which FC was applied with salt (Figs. 4 and 5). Many researchers reported that FC markedly alleviated the detrimental effect of salinity on the germination and growth of various plants^52–54^ and this results are consistent with the findings obtained in the present work. FC have demonstrated its success on salt-induced bulb germination and growth by increasing water uptake (Fig. 4), by stimulating mitotic activity (Fig. 6), by reducing lipid peroxidation (Fig. 9) and by regulating free proline content and antioxidant enzyme activities (Fig. 9). In addition, it may have also performed its success on salt stress by providing the stabilization of cell membranes^55^ or by staging a counter-attack against the abscisic acid (ABA), the internal amount of which increases in the plant in salinity^54,56^.

### Effect of FC on the cytogenetic parameters

The effects of exogenous FC on some cytogenetic parameters (mitotic index/MI, micronucleus/MN frequency, chromosome aberrations/CAs) in the root cells of onion bulbs exposed to salinity are shown in Fig. 6. The MI (7.1 ± 0.8%) in the root of Group III bulbs grown in the medium with FC alone increased by 1.2 fold according to MI (5.9 ± 0.5%) of Group I (control) bulbs grown in tap water medium, whereas their MN frequency and CAs statistically demonstrated similar values as ones of the control group.

The degree of cytotoxicity of a chemical agent can be determined by the change in MI^57^. Salinity stress both reduces the mitotic activity and causes serious damages on the chromosome structure^58,59^. Similar results were also observed in the present study. While MI was 5.9 ± 0.5% in the roots of Group I (control) bulbs grown in tap water medium, this parameter was 2.3 ± 0.3% in the roots of Group II bulbs grown in 0.15 M NaCl medium and decreased by 61% compared to the control. At the same time, this salt concentration (0.15 M) caused a significant increase on the MN frequency and CAs in onion root tips. For instance, MN frequency and CAs in the roots of Group I (control) bulbs grown in tap water medium were calculated as 0.9 ± 0.2% and 1.3 ± 0.7%, respectively, while these parameters in the roots of Group II bulbs grown in 0.15 M NaCl medium showed a statistically significant increase (p < 0.05) and were determined as 9.8 ± 1.6% and 27.2 ± 1.8% (Fig. 6).

On the other hand, 3 µM exogenous FC application to Group IV bulbs grown in 0.15 M NaCl medium significantly increased MI, decreased MN frequency and CAs (p < 0.05). The MI, MN frequency and CAs in the root cells of Group II bulbs in 0.15 M NaCl medium alone were 2.3 ± 0.3%, 9.8 ± 1.6% and 27.2 ± 1.8%, respectively, while these parameters were determined as 5.9 ± 0.6%, 4.3 ± 1.2% and 7.6 ± 1.7% in the root cells of Group IV bulbs in which FC was applied with salt (Fig. 6). These findings are a sign that exogenous FC administration can alleviate salt-induced damage on the mitotic activity and chromosome structure. The present study is the first study to investigate the effects of FC on MN frequency and CAs in the root cells of plants grown under both normal and saline conditions. In addition, it has been found only one study about the effects of FC on the MI of the root tip cells under normal and saline conditions in result of the literature review so far. In the mentioned work, Lutsenko et al.^46^ reported that 5 × 10^–6^ M FC application increased the MI in the root meristematic cells of sorghum seedlings under both normal and saline (0.1 and 0.2 M NaCl) conditions, and this result was similar to our current research findings.

**Figure 7:**
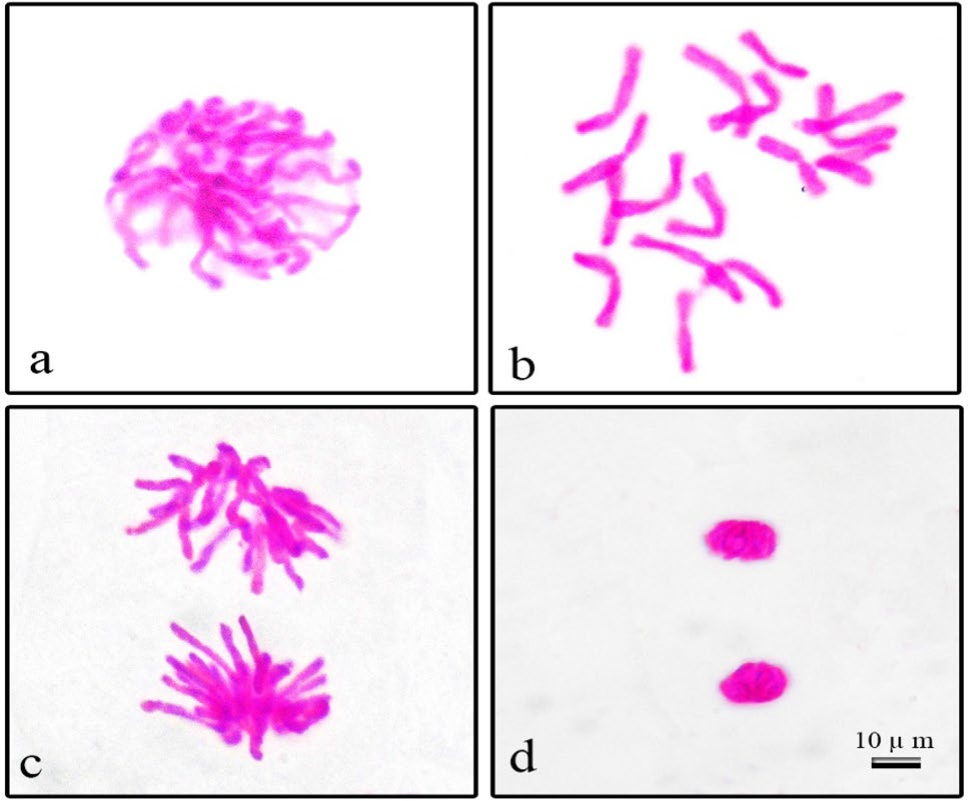
Normal mitosis phases in *Allium cepa* root meristem cells (**a**) prophase (**b**) metaphase, 2n = 16 chromosomes (**c**) anaphase (**d**) telophase. Scale bar = 10 μm.

**Figure 8:**
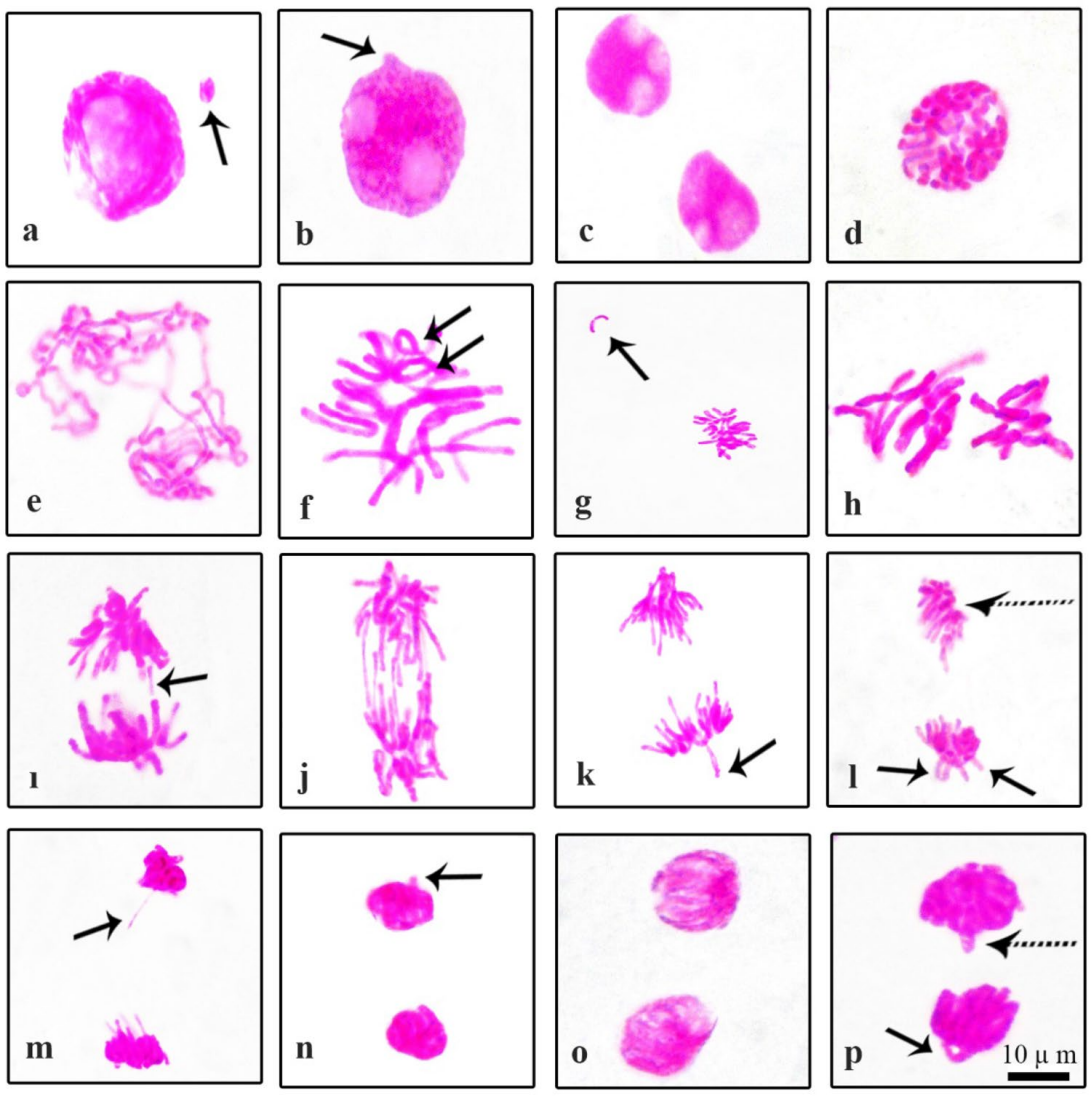
Chromosomal aberrations in *Allium cepa* root tip cells (**a**) micronucleus = arrow (**b**) lobulated nucleus with bud(= arrow) (**c**) several lobulated nuclei (**d**) chained prophase (**e**) irregular prophase (**f**) metaphase with chromosomes encircled(= arrows) (**g**) metaphase with chromosomal loss(= arrow) (**h**) disrupted equatorial plate in metaphase (**i**) anaphase with fragment(= arrow) (**j**) bridges in anaphase (**k**) anaphase with vagrant chromosome(= arrow) (**l**) polar slip (= patterned) at anaphase with vagrant chromosomes(= arrows) (**m**) lagging chromosome in anaphase(= arrow) (**n**) telophase with vagrant chromosome(= arrow) (**o**) polar slip (= patterned) at telophase (**p**) lagging chromosome (= patterned) at telophase with chromosome loop. Scale bar = 10 μm.

Normal mitotic phases observed during microscopic examination of onion root tip preparations are shown in Fig. 7 and abnormal mitotic phases are shown in Fig. 8. Significant abnormalities in the preparations were micronucleus (Fig. 8a), lobulated nucleus/several lobulated nuclei (Fig. 8b,c), chained prophase (Fig. 8d), irregular prophase (Fig. 8e), metaphase with chromosomes encircled (Fig. 8f), metaphase with chromosomal loss (Fig. 8g), disrupted equatorial plate in metaphase (Fig. 8h), anaphase with fragment (Fig. 8i), bridges in anaphase (Fig. 8j), anaphase/telophase with vagrant chromosome (Fig. 8k,l,n), polar slip at anaphase/telophase (Fig. 8l,o), lagging chromosome in anaphase/telophase (Fig. 8m,p). MN occurs as a result of chromosomes or chromosome breaks that remain in anaphase and fail to fuse with both nuclei in telophase^60,61^. Although their incorporation into the nucleus is different, bud and MN formation are morphologically similar^62^. MN and bud formation can lead to loss of genetic material^63^. Watched chromosome fragment shows clastogenic action while chromosome stickiness could be a consequence of inter-chromosomal linkages coupled with excessive formation of nucleoproteins. Chromosome loss is a change that occurs with the malfunction of the mitotic spindle^64^. The vagrant chromosome is derived from the irregularly shaped and unequally sized nuclei of daughter cells with unequal chromosomes^65^. Chromosome stickiness and impaired chromosome segregation can lead to bridge formation in anaphase and cause structural mutations in the chromosome^66^. Lobed nuclei forms when a nuclear poison 214 blocks DNA synthesis in the S phase of the interphase^67^. Lagging chromosomes result due to failure of the chromosomes to become attached to the spindle fiber and to move to either of the two poles^68^. Anaphase and telophase with fault polarization occur as a result of spindle disorders^69^.

### Effect of FC on the biochemical parameters

**Figure 9:**
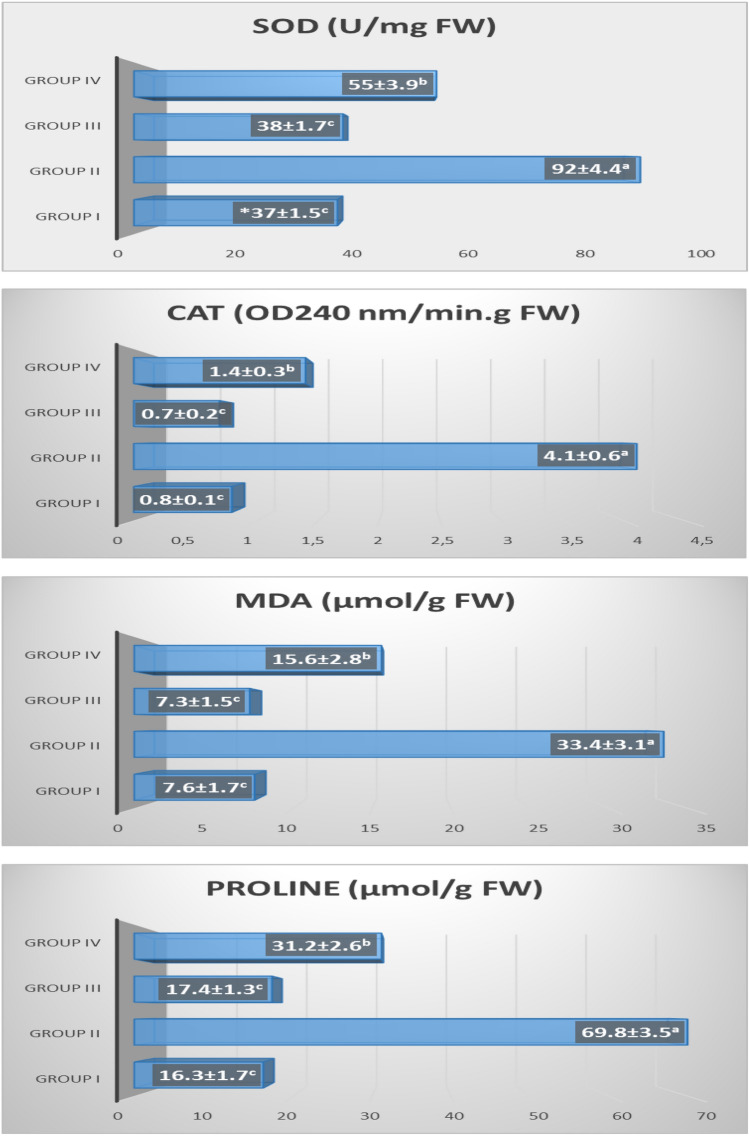
Effect of FC on some biochemical parameters of *Allium cepa* L. Group I (control) was treated with tap water; Group II was treated with 0.15 M NaCl; Group III was treated with 3 µM FC; Group IV was treated with 3 µM FC + 0.15 M NaCl. The ± symbol indicate the standard deviation (SD).

Free radicals or reactive oxygen species (ROS) are cytotoxic molecules that can damage important cellular materials such as DNA, protein, carbohydrates and lipids. These highly reactive molecules also regulate the expression of defense systems-related genes as intermediate signaling molecules^70,71^. Plants contain antioxidant enzymes such as superoxide dismutase (SOD) and catalase (CAT), which have scavenging effects to prevent damage to cellular structures by ROS^72,73^. It was determined that SOD (38 ± 1.7 U/mg FW) and CAT (0.7 ± 0.2 OD240 nm/min g FW) enzyme contents in the roots of Group III bulbs grown in FC medium alone were statistically similar to those (SOD 37 ± 1.5 U/mg FW; CAT 0.8 ± 0.1 OD240 nm/min g FW) of Group I, which was grown in tap water medium and the control group (Fig. 9). This data clearly showed that exogenous FC application did not cause an additional ROS formation in onion roots.

Neverthless, NaCl stress caused a significant increase in SOD (92 ± 4.4 U/mg FW) and CAT (4.1 ± 0.6 OD240 nm/min g FW) enzyme activities in the roots of Group II bulbs by 2.5 and 5.1 times, respectively, compared to those (SOD 37 ± 1.5 U/mg FW; CAT 0.8 ± 0.1 OD240 nm/min. g FW) of Group (control) I (Fig. 9). SOD and CAT antioxidant enzyme contents were found to increase in many plant species exposed to salinity stress^74–76^. The increase in SOD, CAT, MDA, MN frequency and CAs in the roots of Group II bulbs is a reliable indicator of NaCl-induced ROS formation and oxidative stress (Figs. 6 and 9). On the other hand, 3 µM exogenous FC application to Group IV bulbs grown in 0.15 M NaCl medium significantly reduced SOD and CAT levels (p < 0.05). The SOD and CAT amounts in the root cells of Group II bulbs in 0.15 M NaCl medium alone were 92 ± 4.4 U/mg FW and 4.1 ± 0.6 OD240 nm/min g FW, respectively, while these parameters were determined as 55 ± 3.9 U/mg FW and 1.4 ± 0.3 OD240 nm/min g FW in the root cells of Group IV bulbs in which FC was applied with salt (Fig. 9). The fact that FC applied externally to Group IV bulbs exposed to salinity reduces SOD and CAT contents is an important sign that ROS is swept away and salt tolerance is achieved.

Lipid peroxidation (LP) is the reaction of oxidative degradation of polyunsaturated lipids by ROS. As an indicator of LP, determination of malondialdehyde (MDA) levels is made by using spectrophotometric methods^77,78^. It was determined that MDA (7.3 ± 1.5 µmol/g FW) content in the roots of Group III bulbs grown in FC medium alone was statistically similar to that (7.6 ± 1.7 µmol/g FW) of Group I, which was grown in tap water medium and the control group (Fig. 9). This data clearly showed that exogenous FC application did not cause an additional damage in membranes of the root cells.

Neverthless, NaCl stress caused a significant increase in MDA (33.4 ± 3.1 µmol/g FW) amount in the roots of Group II bulbs by 4.4 times, approximately, compared to that (7.6 ± 1.7 µmol/g FW) of Group I/the control (Fig. 9). Salt stress showed a destructive damage on the cell membranes by increasing the MDA content statistically (p < 0.05). MDA content, which is an indicator of LP was found to increase in many plant species such as tomato^79^, sweet pepper^80^, mint^75^ and mung bean^81^ exposed to salinity stress. On the other hand, 3 µM exogenous FC application to Group IV bulbs grown in 0.15 M NaCl medium significantly reduced MDA level (p < 0.05). The MDA amount in the root cells of Group II bulbs in 0.15 M NaCl medium alone was 33.4 ± 3.1 µmol/g FW, while this parameter was determined as 15.6 ± 2.8 µmol/g FW in the root cells of Group IV bulbs in which FC was applied with salt (Fig. 9). The fact that FC applied externally to Group IV bulbs exposed to salinity reduces MDA content is an important sign that ROS is swept away, oxidative stress is suppressed and salt tolerance is achieved.

Proline (PR), one of the amino acids that make up proteins, is an osmolyte commonly produced in plants exposed to various environmental stresses such as salinity^82^. This amino acid, which is synthesized through glutamate in plants, serves to maintain osmotic potential and turgor^83,84^. However, it also undertakes the task of protecting cells by stabilizing cell membranes and proteins during dehydration^85,86^.

It was determined that free PR (17.4 ± 1.3 µmol/g FW) content in the roots of Group III bulbs grown in FC medium alone was statistically similar to that (16.3 ± 1.7 µmol/g FW) of Group I, which was grown in tap water medium and the control group. Neverthless, NaCl stress caused a significant increase in free PR (69.8 ± 3.5 µmol/g FW) amount in the roots of Group II bulbs by 4.3 times, approximately, compared to that (16.3 ± 1.7 µmol/g FW) of Group I/the control (Fig. 9). It has been reported in previous studies that PR amino acid accumulates in plants exposed to salt stress^87,88^ and salt tolerance of plants increases^89,90^. However, a negative correlation between abiotic stress tolerance and accumulation of free PR has also been reported^91,92^. On the other hand, a positive correlation between MDA and PR accumulation^76^ was once again confirmed by this study. These data are a strong indication that PR helps scavenge salt-induced ROS and protect cells from oxidative damage^93^.

On the other hand, 3 µM exogenous FC application to Group IV bulbs grown in 0.15 M NaCl medium significantly reduced free PR level (p < 0.05). The free PR amount in the root cells of Group II bulbs in 0.15 M NaCl medium alone was 69.8 ± 3.5 µmol/g FW, while this parameter was determined as 31.2 ± 2.6 µmol/g FW in the root cells of Group IV bulbs in which FC was applied with salt (Fig. 9). The reduction of free PR content of FC in the roots of Group IV bulbs exposed to salinity may allow the expression of new proteins associated with stress tolerance. Because, Khedr et al.^94^ reported that PR amino acid increased the protein content in sea daffodil (*Pancratium maritimum* L.) plant exposed to salt stress. This is the first study to examine its effects on antioxidant enzyme (SOD and CAT) activity, LP and free PR content of externally applied FC to plants grown in a stress-free medium or exposed to salt stress. Therefore, the data obtained from this study are very important.

### Effect of FC on the anatomic parameters

**Figure 10:**
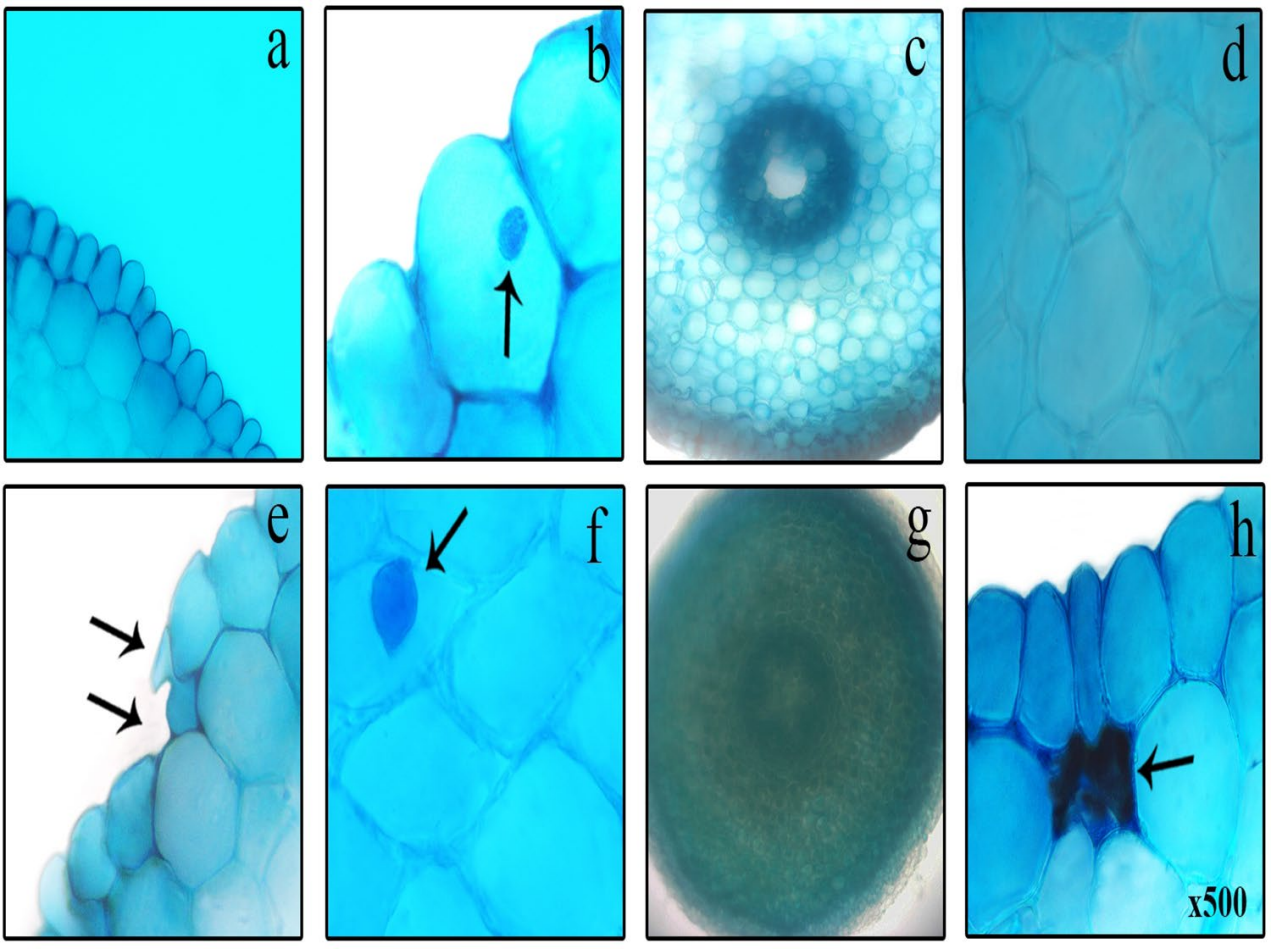
Anatomic damages induced by NaCl in root tip cells (**a**) healthy appearance of epidermis cells (**b**) healthy appearance of cell nucleus (oval) (= arrow) (**c**) normal appearance of transmission tissue (**d**) healthy appearance of cortex cells (**e**) epidermal cell injury (= arrows) (**f**) giant cell nucleus (= arrow) (**g**) indistinct transmission tissue (**h**) necrotic areas (= arrow).

Root is an organ that usually grows into the soil in developed plants adapted to land life. It is an organ that is frequently exposed to environmental stresses and toxic agents because it is in contact with the soil. As a result of this exposure, serious changes and damages occur in the anatomical structure of the root. The anatomical damages caused by salinity stress in the roots of onion bulbs and the protective role of FC against these damages are shown in Table 1 and Fig. 10.

**Table 1:** Determination of the damages observed in the root anatomical structures of bulbs.

Experimental groups	ECI	GCN	ITT	NA
Group I/control	−	−	−	−
Group II	+++	++	+++	+++
Group III	−	−	−	−
Group IV	+	+	+	+

ECI epidermal cell injury, GN giant cell nucleus, ITT indistinct transmission tissue, NA necrotic areas, (−) no damage, (+) little damage, (++) moderate damage, (+++) severe damage.

As a result of examination with the help of light microscopy of the preparations prepared from the roots of Group I (control) bulbs grown in tap water medium and Group III bulbs grown in 3 µM FC medium alone, no anatomical damage was found. On the contrary, in the preparations prepared from the roots of Group II bulbs grown at 0.15 M salinity alone and Group IV bulbs treated with 3 µM FC together with salt, epidermal cell injury (Fig. 10e), giant cell nucleus (Fig. 10f), indeterminate vascular tissue (Fig. 10g) and necrotic areas (Fig. 10h) were observed.

These damages and changes observed in root anatomical structure are the result of defense mechanisms developed by cells and tissues to reduce the severity of salinity. For example, plants exposed to environmental stresses or hazardous chemicals develop mechanisms that alter the anatomical structure, such as reduced substance transport and indistinct vascular tissue, to mitigate the damage caused by these stresses and chemicals^95^. Epidermal cell damage is a sign that high salinity disrupts cell wall integrity. Shape change may occur in response to cellular changes in the nucleus, which is normally spherical or elliptical in appearance. In addition, disruptions in DNA double helix structure, DNA volume and nuclear protein concentration can lead to abnormalities in the volume and shape of the cell nucleus^96,97^.

On the other hand, 3 µM exogenous FC application to Group IV bulbs grown in 0.15 M NaCl medium significantly reduced the severity of root anatomical structure damage (Table 1). This is the first study in the literature to investigate the effects of exogenous FC on the root anatomical structure of plants exposed to salt stress.

## Conclusion

In this study, the role of FC, a phytotoxin, in mitigating the negative effects of salinity in onion plant, which is consumed as an important food source all over the world, was investigated by considering physiological, cytogenetic, biochemical and anatomical responses. When the new scientific data obtained from the current study are evaluated, it can be said that FC applied externally at the appropriate dose and time increases the tolerance to salinity in the onion plant. In addition, the positive effects of FC in the germination phase of onion bulbs give the impression that it can play a role as a growth regulator in growth-development regulation.

## Data Availability

The datasets used and/or analysed during the current study available from the corresponding author on reasonable request.
